# The Influence of Fiber Cross-Section on Fabric Far-Infrared Properties

**DOI:** 10.3390/polym10101147

**Published:** 2018-10-14

**Authors:** Yifei Tao, Tenghao Li, Chenxiao Yang, Naixiang Wang, Feng Yan, Li Li

**Affiliations:** 1 Institute of Textiles and Clothing, The Hong Kong Polytechnic University, Hung Hom, Kowloon, Hong Kong, China; yifei.tao@polyu.edu.hk (Y.T.); chenxiao.yang@connect.polyu.hk (C.Y.); 2 Department of Applied Physics, The Hong Kong Polytechnic University, Hung Hom, Kowloon, Hong Kong, China; morganhoho@163.com (T.L.); wangnaixiang.hust@gmail.com (N.W.); apafyan@polyu.edu.hk (F.Y.)

**Keywords:** far-infrared radiation, absorption and emission, cross-section, polyamide fiber

## Abstract

Far-infrared radiation (FIR) possesses various promising properties that are beneficial to an individuals’ health. Exploring the interaction between fiber shapes and FIR performance is thought to be a significant means to develop highly-efficient FIR textile products. In this study, a non-additive triangular polyamide (PA) fiber showed excellent FIR properties in both theoretical simulation and experimental verification aspects. The triangular PA fiber affords a higher probability to facilitate large optical path difference, improving both FIR absorption and emission. Textiles woven with the specific triangular PA fiber achieved a remarkable emissivity of 91.85% and temperature difference of 2.11 Celsius, which is obviously superior to the reference circular fiber (86.72%, 1.52 Celsius). Considering the low cost, environmental stability, facile fabrication, as well as being environmentally friendly, this non-additive triangular PA fiber has great potential for high-performance and cost-effective FIR textiles in the future.

## 1. Introduction

Far-infrared radiation (FIR), as an environmentally friendly and sustainable energy source, can be utilized in many functional textiles to provide not only thermal comfort but also thermal therapy [1,2,3,4]. According to the annual research and consultation report, a huge commercial market of FIR textile products is emerging, which is expected to explode in the near future. Specifically, with the improvement of public health awareness and consumption capacity, the global FIR textile market size, as well as its business net profit margin, is set to demonstrate remarkable growth continuously [5]. Moreover, as an FIR textile product, Celliant^®^ has been classified as a medical device and general wellness product by the U.S. Food and Drug Administration (FDA), which will promote wide commercial cooperation with numerous brands in different areas. Furthermore, on tracking the sales volume in different continents, Asia’s FIR textile market share is found to be almost blank, thus, the FIR textile industry will obviously embrace broad development prospects.

Due to FIR’s excellent penetrability, energy transmission is purely from light energy to heat energy in human skin. The thermal effect within deep tissues can expand the blood vessels and promote blood microcirculation to eliminate metabolic toxins and waste [6,7]. Therefore, FIR textiles are always considered as an integration of therapeutic and thermal-protective segments, which have been widely used for various kinds of healthcare applications, such as warmth retention [8,9,10], relief from inflammation [11,12], wound healing [13,14], weight loss [15,16] and relief from aching pains [17,18]. Similarly, considering its physical therapy efficacy, the general requirements for FIR products in European and American countries are to assess a subjects’ blood oxygen content and flow velocity through a human wear trial over weeks or months. However, several relative systematic and quantitative evaluation standards have already been established in Asia, based on the principle of emissivity and temperature difference. For instance, Japan Far Infrared Rays Association (JIRA) product identification technical benchmark (Japan), FTTS-FA-010: Specified requirements of far infrared textiles (Taiwan) and GB/T 30127-2013: Textiles-Testing and evaluation for far infrared radiation properties (Mainland of China), are the most typical representatives, of which the GB/T 30127-2013 National Standard will be adopted here to examine the proposed FIR textiles.

Currently, the normal technology for realizing far-infrared textiles is to introduce ceramic powders, including magnesium oxide, silicon oxide, zirconium carbide, and germanium compounds [19,20,21,22,23], in the textile through coating, spraying, laminating, impregnating, covering, and dipping processes [11]. In general, melt-spinning and after-treatment methods are primary approaches used in commercialized FIR textile products. For melt-spinning, ceramic nanoparticles are mixed with the raw polymer masterbatch prior to fabricating synthetic FIR fibers. In the after-treatment process, far-infrared adhesives are fixed onto the surface of a substrate to endow the composites with FIR properties. The weaving unit FIR fiber is thought to be a critical factor to affect FIR textile performance. The basic requirement for a desirable FIR fiber includes weavability, high emissivity, and stability. Optimizing the density and dispersity of far-infrared powders within the spinning matrix to maximize its FIR emissivity has been considered a promising approach for exploiting innovative FIR fibers. Impressively, a positive sputtering means for manufacturing far-infrared ceramic-coated substrate has been reported [24]. In addition, another crucial approach is to seek higher efficient FIR raw materials and improve their corresponding performance subsequently. Hu et al. demonstrated that a graphene–polyurethane compound coating could enhance the FIR emissivity of cotton fabric up to 0.911 [25]. Hsu et al. developed a superior dual-mode textile with a three-dimensional hierarchical structure to achieve both a radiative heating and cooling function without any additional energy input [26]. Furthermore, as a widely effective chemical fiber, PA fiber has been proven to possess desirable FIR properties [27], but few attempts referring to the interaction between the cross-section shapes and FIR performance have been investigated.

**Scheme 1:**
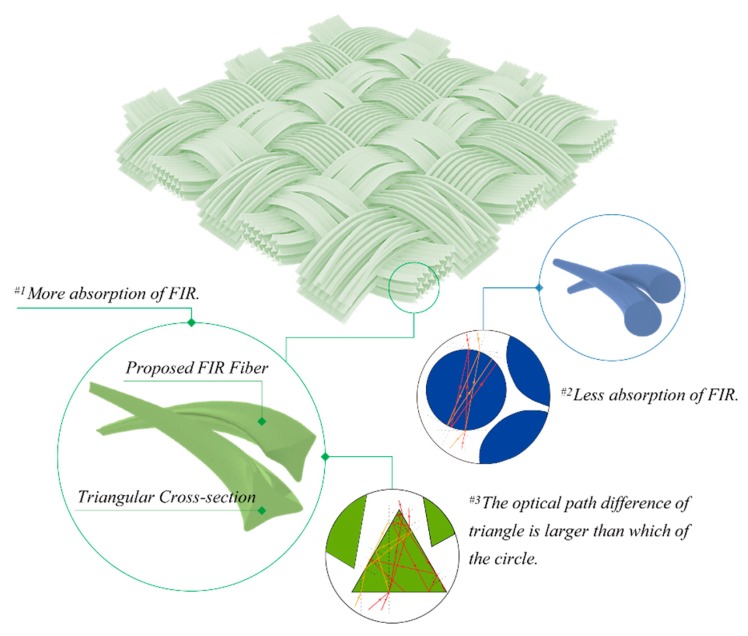
Schematic illustration of the optical path between triangular fiber (left) and circular fiber (right). FIR = Far-infrared radiation.

In this work, we propose a non-additive triangular PA fiber to explore the effect of fiber shape on FIR performance, which is expected to develop a convenient and efficient FIR textile technology. Textiles woven with the specific profiled fiber presented high emissivity, excellent environmental stability, and low-cost. To highlight the shape advantage of the proposed triangular PA fiber, another circular PA fiber was taken as a control sample. As well illustrated in Scheme 1, different fiber shapes can bring completely different optical paths. Triangular fibers possess a higher probability to generate a large optical path difference, in turn, to accelerate both FIR absorption and emission. The overall research approaches are divided into theoretical simulation and experimental assessment stages, wherein, the former aims to derive the potential law or appropriate calculation formula based on existing physical laws, and the latter is conducted to verify the underlying relationship between the FIR performance and cross-sectional shapes.

## 2. Experimental Section

### 2.1. Materials

All of the PA fibers with different parameters were purchased from Yiwu Huading Nylon Co., Ltd. (Yiwu, China). Elastic yarns were bought from Hong Kong Kam Hing Threads Ltd. (Hong Kong, China). Table 1 shows the detailed specifications of the above yarns, such as the cross-sectional shapes, glossiness, draft degree, and filaments.

### 2.2. Preparation of Fabrics

PA far-infrared fabrics were manufactured by knitting with a CMS822 automatic flat knitting machine of the STOLL Company (Reutlingen, Germany), and the fabrics were knitted with PA and elastic yarns. For the STOLL flat knitting machine, the density of fabric was mainly controlled by the NP value, which is a machine parameter to adjust the needle’s downward position of loop formation. In this experiment, the NP value was fixed to 9.5, which is a normal value for full-needle fabric on a flat knitting machine. In consequence, the density of the FIR fabrics was 9.26 courses per cm and 8.64 wales per cm in the wales and course directions, respectively. In order to improve the precision and reliability of the experimental results, elastic yarns were employed to increase the tension of fabric during the knitting process. As a result, the prepared far-infrared fabric was uniform and with a lower porosity.

### 2.3. Fabrics Characterization

Optical microscopy (LeicaDM4P, Leica Microsystem, Wetzlar, Germany) was used to investigate the morphology of yarns and as-prepared fabrics. The far-infrared performance of PA fabrics was qualitatively characterized by a Fourier Transform Infrared Spectrometer (FT-IR, Bruker tensor27, Billerica, MA, USA) recorded from 400 to 4000 cm^−1^ in attenuated total reflection (ATR) mode using a Ge crystal-plate, and scanning 100 points for each sample to take the average value for the FT-IR spectrum. Infrared thermal images were performed through the FLIR system (Fluke568, Fluke Corporation, Everett, WA, USA) on a constant metal heating stage under thermal balance status. The emissivity and temperature differences of fabrics were carried out with far infrared equipment (DR915G & DR915W, Darong Textile Instrument Limited, Wenzhou, China) based on the national standard method (GB/T 30127-2013). All of the fabric samples were washed according to the AATCC 135-2012 standard before testing.

### 2.4. Results and Discussions

**Figure 1:**
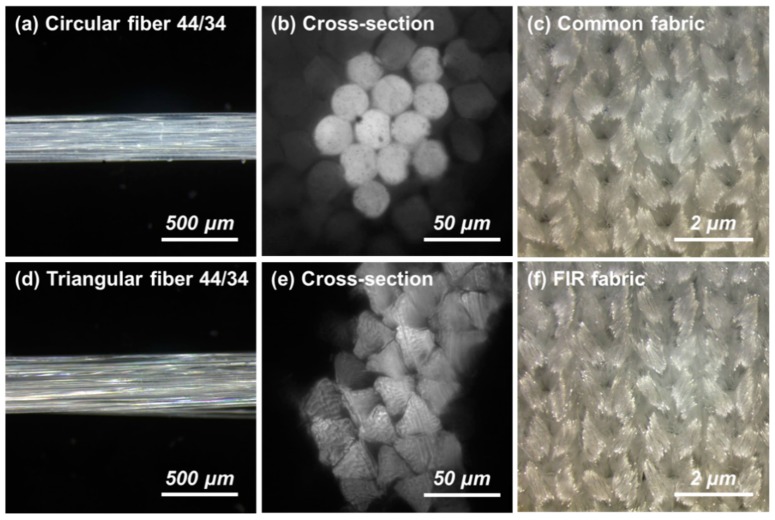
Optical morphologies of (**a**) surface of circular fiber; (**b**) cross-section of circular fiber; (**c**) surface of full needle common fabric with circular fibers; (**d**) surface of triangular fiber; (**e**) cross-section of triangular fiber; (**f**) surface of full needle far-infrared radiation (FIR) fabric with triangular fibers.

The surface morphology of the fabric samples and cross-sectional shapes of the PA yarns were investigated by optical microscopy, as shown in Figure 1. For circular and triangular PA yarns, based on a single variable principle, except for the cross-sections being different from each other, the glossiness (bright), 44 dtex with 34 filaments, and fully drawn yarn (FDY) parameters are all the same. Meanwhile, in order to improve the strength and performance of FIR fabrics, six PA yarns were first bundled and sewn into a single yarn, and then the lined-up yarn was knitted with elastic yarn to constitute the whole FIR fabric, wherein the weavability of the PA yarn still maintained normality. As can be seen from the microstructure of both FIR fabrics, the porosity of fabric samples is almost the same, thus, the influence on FIR performance differences of far-infrared radiation transmission is negligible in this study. In addition, although the delustering agent content of the triangular PA yarn is the same as for circular PA yarn, the glossiness is slightly different between the FIR fabric and common fabric. Therefore, to a certain extent, the cross-sectional shapes of a single fiber can also affect the spread of light, whether it is visible light or invisible far-infrared radiation.

**Figure 2:**
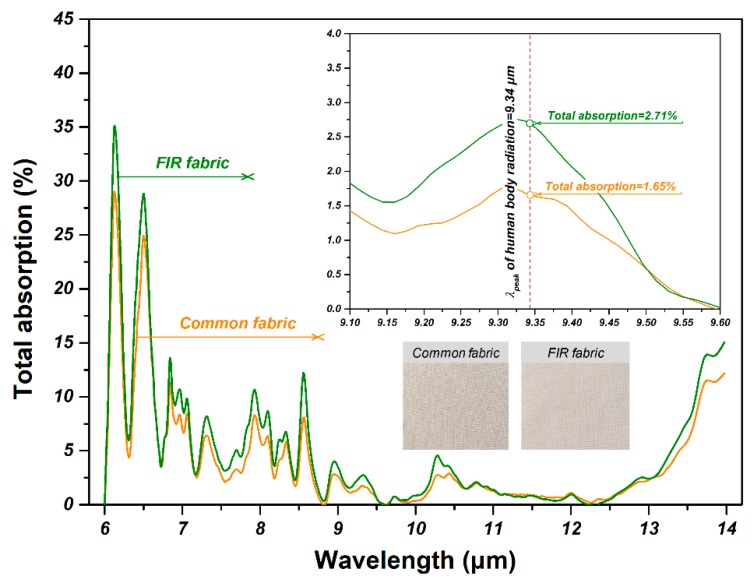
The FT-IR spectra of common fabric and FIR fabric in the range of 6 to 14 μm.

FT-IR spectra were performed to make a qualitative analysis of the far-infrared performance between the common fabric and FIR fabric, as shown in Figure 2. It is of note that the overall absorption of the FIR fabric is significantly higher than the common fabric between the wavelength of 6–14 μm (life-rays). Particularly, at the peak wavelength (9.34 μm) of human body radiation, the average absorption of FIR fabric is estimated at about 2.71%, which is clearly greater than that of the common fabric (1.65%, seen in the magnified image). At the same time, the relative deviation of absorption between the triangular and common fabrics is up to 64.24%, therefore, the triangular shape can obviously induce a longer propagation distance of FIR inside the fiber than the circular counterpart, which can thus enhance FIR absorption of the resulting fabrics.

**Figure 3:**
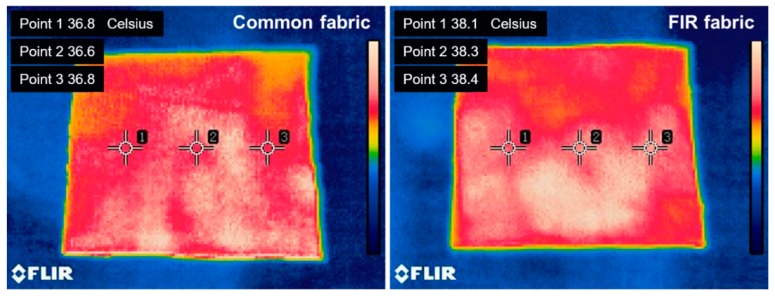
Thermal images of common fabric (**left**) and FIR fabric (**right**) under the thermal equilibrium state.

In addition to indirectly assessing FT-IR, the intuitive evaluation of infrared imaging was also applied to both prepared fabrics, which were in thermal equilibrium with the constant heating stage before imaging. Figure 3 presents the thermal images of the common fabric and FIR fabric, respectively. For the FIR fabric, the measured point temperature was almost uniformly distributed and the average value is close to 38.27 Celsius. In contrast, the thermal image showed an average temperature of common fabric is 36.73 Celsius, which is 1.54 Celsius lower than that of the FIR fabric. Although both of the fabrics have the same temperature, lower emissivity results in less far infrared radiation so that the IR camera captures different temperature variation. In accordance with Kirchhoff’s law, the emissivity and absorptivity have the same variation trend under the thermal equilibrium state, thus, the FIR fabric presents a relatively high temperature in the thermal image.

**Figure 4:**
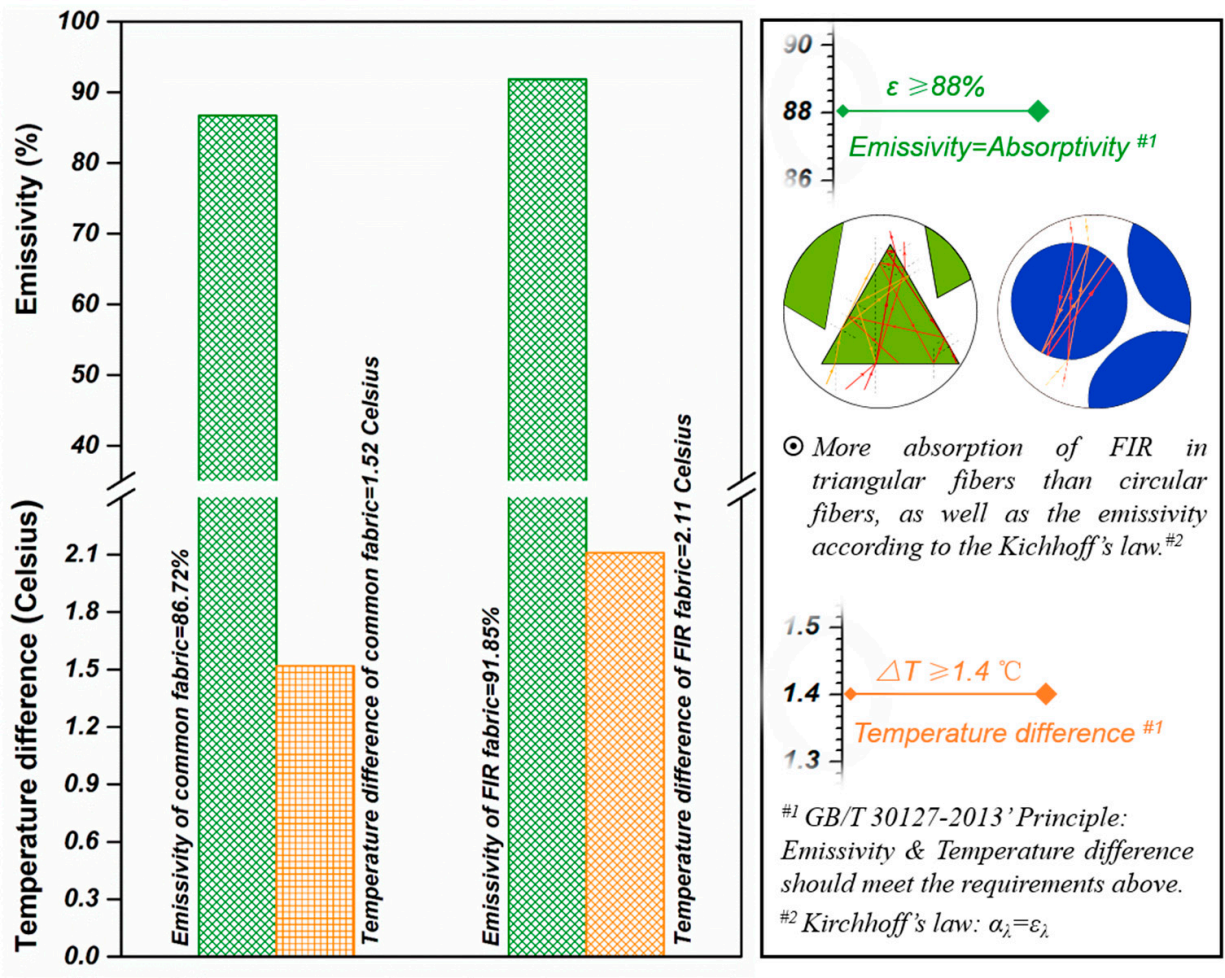
The emissivity and temperature difference of common fabric and FIR fabric. *α* means absorptivity, *ε* means emissivity.

To further assess its potential as a far infrared textile in the current market, the far infrared radiation properties of FIR fabric were also conducted according to the national standard method, as well as the common fabric being studied for comparison. As shown in Figure 4, the FIR fabric delivers emissivity of 91.85%, and temperature difference of 2.11 Celsius, which is ascribed to the enhanced far-infrared radiation absorption efficiency. Compared with the common fabric, the far infrared radiation properties of FIR fabric demonstrate an obvious advantage with increases in emissivity and temperature difference behavior of 5.13% and 0.59 Celsius, respectively. Moreover, as stated in the national standard of the far infrared textiles, the emissivity and the temperature difference should not be lower than 88% and 1.4 Celsius for a satisfactory commercial textile, therefore, the FIR fabric is an effective far infrared radiation emitter, which is also suitable for personal thermal and physiotherapy textiles. Furthermore, without any far infrared additives in the production process it also provides more powerful competitiveness in the conventional far infrared industry, as well as being in line with the development trend of environmentally friendly textiles.

## 3. Theoretical Analyses and Numerical Simulations Section

From the above experimental studies, it is evident that FIR performance can be improved through tailoring the fiber shape. More detailed theoretical investigations are performed in this section, which provides deeper understanding on the underlying mechanisms. The physical principle is first explained, and simulations are then illustrated with the model and the method. Finally, the simulation results and analyses are described on a single fiber, the fibers with a simplified fully-random arrangement, and the fibers with a practical tight arrangement.

### 3.1. Physical Principle

The FIR performance, physically measured as the absorption (or according to Kirchhoff’s law, the emissivity at the same temperature), is not only dependent on the material itself, but also the fibers’ structure and their arrangement in the yarn. In the point-view of geometric optics, the light rays generally experience larger optical path difference and more multiple scattering in the yarn with triangular fibers (see Scheme 1), which then contribute to the overall absorption enhancement compared to the circular one. This rough physical interpretation is strengthened by the simulations and analyses below. It is revealed that there is an absorption fluctuation with different incident angles in the triangular fiber, and the specific arrangement of these fibers in the yarn assures the overall enhancement of FIR performance, by considering the effective absorption cross-section.

**Figure 5:**
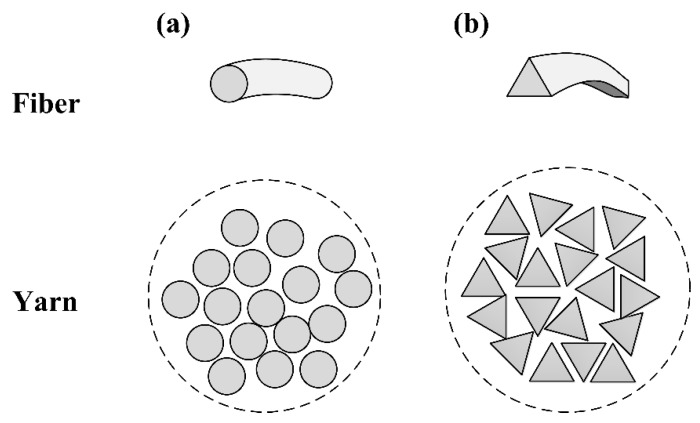
The fibers in the fabric are sketched, including (**a**) circular and (**b**) triangular shapes. The simulations and analyses are performed for the cases of a single fiber to an aggregate of fibers (e.g., yarn).

In the simulations and analyses, both the triangular and circular fibers are considered. The angle-dependent absorption of a single fiber is first investigated, and the arrangement effect is then analyzed in an aggregate of fibers (e.g., yarn), as shown in Figure 5.

### 3.2. Simulation Model

As shown in Scheme 1, it can be seen that the fibers are sewn into yarns in a well-defined style, however, there is relatively-random rotation for a particular fiber along its longitudinal direction. It is also recognized there is a full-angle incidence from the outside irradiation in the general cases. In this regard, the absorption of a single fiber is first characterized in a simplified 2-dimensional cross-section, with respect to each different incident angle. Subsequently, based on the single-fiber results, the performance of the yarn is then further analyzed. Particularly, the absorption cross-section is applied, which offers a direct quantitative comparison between different types of fibers.

**Figure 6:**
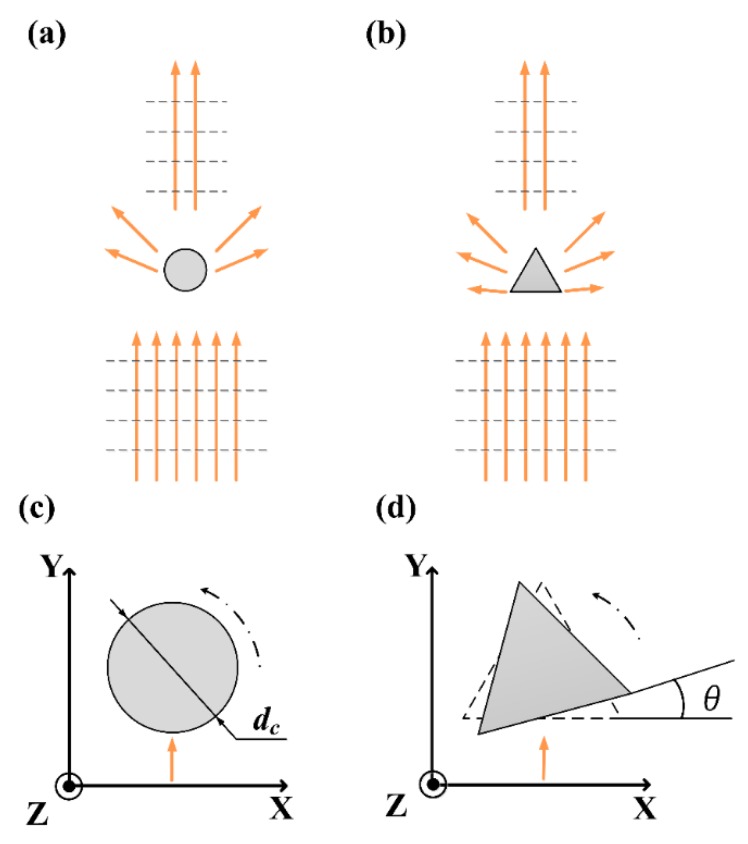
The models of fibers in (**a**,**c**) circular and (**b**,**d**) triangular shapes are presented. The light propagation is sketched in (**a**,**b**), while the coordinate is defined in (**c**,**d**). The yellow arrows denote the propagation light rays, which inject onto the fiber and are then absorbed, scattered or transmitted forward. The black dashed lines indicated the plane-wave field.

The 2-dimensional model is applied in the present simulations, where the single fiber is simplified as an infinitely-long straight rod. Cross-sections for circular and regular-triangular shapes are considered. The light propagation is illustrated in Figure 6a,b for each shape, where a plane wave is used as the light source. Both the scattering and absorption processes take place when the incident plane wave encounters the fiber. By measuring the output, scattered and incident field, both the absorption and scattering can be characterized as values of absorption cross-section and scattering cross-section, respectively [28].

In practice, the light source has a broad incident angle distribution, and can be modeled by either varying the incident angle of the plane wave or by rotating the fiber geometry. The latter was implemented in the simulations, and the coordinate is defined in Figure 6c,d, for the circular and the triangular shapes, respectively. In the O-XYZ system, the fiber is aligned along the Z direction, and the incident light wave propagates along the Y direction. In geometry, a circular shape is rotationally symmetric about the center axis for any angle, however, for the regular-triangular shape, its rotation angle *θ* is defined as shown in Figure 6d, and in particular, it is only necessary to consider the first period with *θ* ranging from 0 to 60 degrees. It is also of note that both of the polarizations (P-wave and S-wave) are considered in the simulations, respectively.

Meanwhile, for comparison of the absorption between circular and regular-triangular shapes, the same area of the cross-section is postulated, namely, the circum-diameter of regular-triangular $d_{t}=\sqrt{4\pi \sqrt{3}/9}d_{c}$, and then the characteristic diameter of circular *d_c_* is set as 20 μm. Besides, according to Wien’s law, the peak wavelength *λ_peak_* of human body radiation is estimated as 9.34 μm, thus a wavelength of 9.34 μm is considered in the simulations [29]. Then the optical constants of the PA fibers are approximated from the experimental measurements, with a refractive index of about 1.54, and extinction coefficient of around 0.05 [30]. The extinction coefficient is extracted approximately from the measured transmittance spectrum, neglecting the relatively small contribution from the reflection [8].

### 3.3. Simulation Method

With the aforementioned models, the finite-difference-time-domain (FDTD) method is adopted in the simulations on the platform of Lumerical’s FDTD Solutions. In the simulation setup, the total-field scattered-field (TFSF) source is used as the light source, with a wavelength of 9.34 μm. Two transmittance box analysis groups, which can measure the optical power flow through the volume, are used to determine the absorption and the scattering cross-sections, respectively. The simulation time is set at a long enough value of 3000 fs, with a time monitor to confirm the time convergence, and Perfectly Matched Layer (PML) is used as the boundary condition.

### 3.4. Simulation Results for a Single Fiber

**Figure 7:**
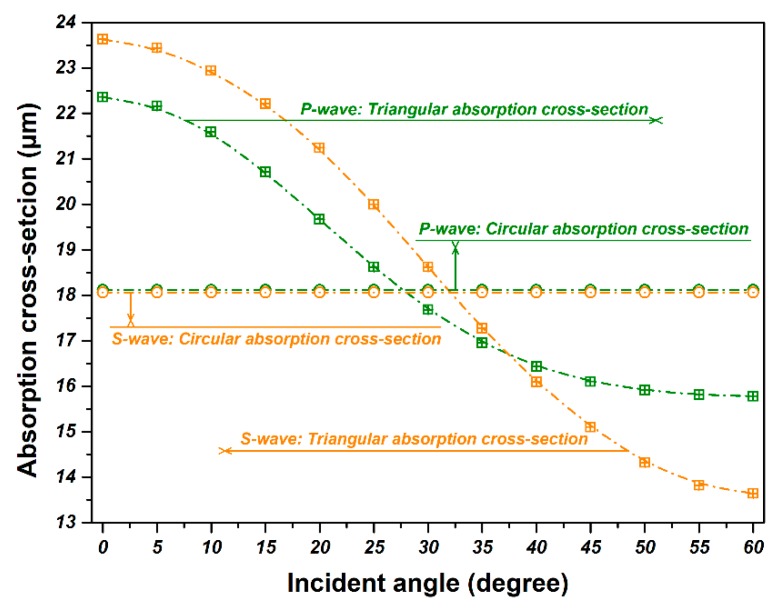
The absorption cross-section of circular and triangular fibers at different incidents. The characteristic diameter *d_c_* is 20 μm and the wavelength 9.34 μm.

The absorption cross-sections of circular and triangular shapes with typical size *d_c_* of 20 μm at the peak wavelength of 9.34 μm for different incident angle are compared in Figure 7. The absorption cross-section of the circular shape (*σ_c_*) is uniform for any incident (rotation) angle, while a periodic variation occurs for the absorption cross-section of the triangular shape (*σ_t_*). Particularly in the period of 0–60 degrees, there is approximately cosinoidal fluctuation for *σ_t_* as a function of the incident angle.

### 3.5. Simulation Results for Simplified Fully-Random Arrangement

**Figure 8:**
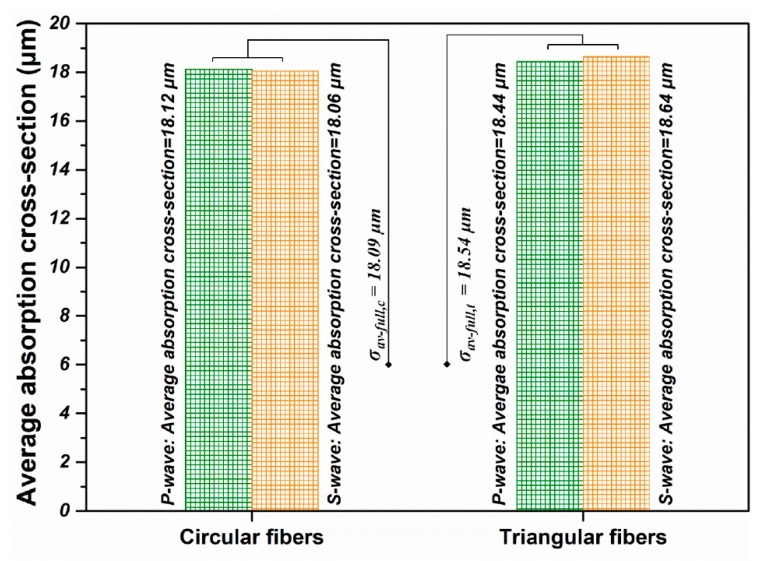
The comparison of average absorption cross-section between the circular and triangular fibers, with the fully-random arrangement.

In a fully-random arrangement, fibers can rotate through the full angle of 0–360 degrees along their own longitudinal directions, and the probability for the occurrence of each angle is assumed to be uniform. Particularly, for the triangular and circular fibers, a range of 0–60 degrees is sufficient for calculations due to the aforementioned periodicity. The mean absorption cross-section of the full-range incident angles is denoted as *σ_av-full_*. In Figure 8, the comparison of *σ_av-full_* between the circular (*σ_av-full,c_*) and triangular (*σ_av-full,t_*) fibers is shown, accounting for both the cases of P-wave and S-wave polarization. Meanwhile, the relative deviation of the absorption cross-section is also presented by calculating *δσ_av-full_* = (*σ_av-full,t_* − *σ_av-full,c_*)/*σ_av-full,c_*. It can be seen that there is a slight increase in the triangular shape when compared to the circular one, with *δσ_av-full_* of about 2.5%.

### 3.6. Simulation Results for the Practical Tightly-Aligned Arrangement

**Figure 9:**
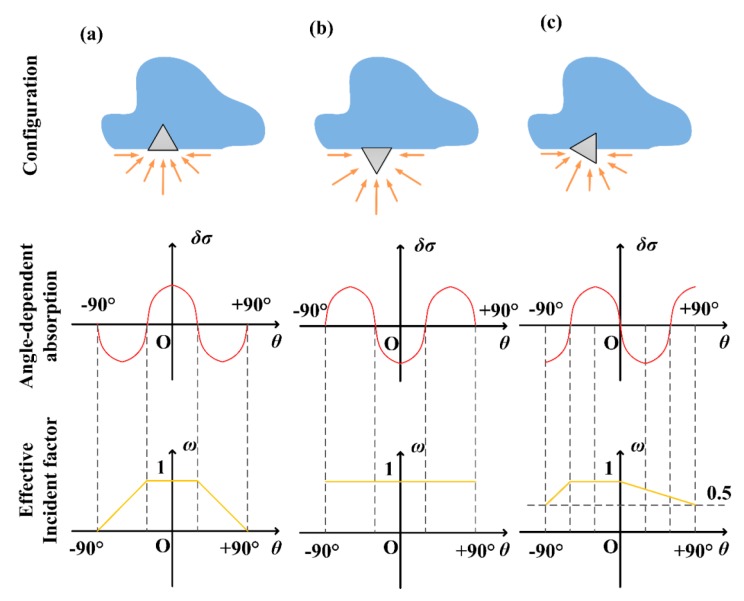
Three typical configurations for the triangular shape at the surface of a yarn are shown, with (**a**) planar, (**b**) tip, and (**c**) half-tip, respectively. The blue part in each configuration stands for the body of a yarn.

However, for the yarn with tightly-aligned fibers in practice, the above full angle averaging method is biased to offer a proper explanation. More rigorous analyses are proven as follows, in the cases where the outer surface of the yarn is smoother, and the fibers inside the yarn are tightly-aligned with a bounded rotation. In a first-order simplification, only the fibers at the outer surface of the yarn are considered while the effect of the inner fibers is neglected, since the outer ones directly encounter the incident wave and naturally present the strongest absorption. As shown in Figure 9, the surface fibers are generally not exposed to the incident wave from all directions as some portions are bared inside the yarn. Thus, only those exposed portions are accounted for, and the effective absorption cross-section *σ_eff_* will be calculated instead of the mean absorption at full angles (*σ_av-full_*). In allusion to the circular shapes, the effective absorption cross-section is nearly the same as *σ_c_*. In contrast, the triangular shapes have a different effective absorption cross-section from the simple full-angle one, resting with the position and rotation. The three typical configurations are shown in Figure 9, including the planar, tip, and half-tip situations.

For each specific configuration, only the exposed portions receive the incident wave. There are non-uniform distributions for both the absorption cross-section and the incident wave amount. Similar to the fully-random arrangement, the relative deviation of the absorption cross-section *δ_σ_* is assumed to be a cosinoidal waveform (red curves in Figure 9). The waveform is assumed to have zero mean value, with the original mean value *δσ_av-full_* of 2.5% (seen in Figure 8) extracted for the simple calculations. The amplitude *δσ_av-amp_* is calculated as the average of *δσ_av-up_* (*δσ_av_* for angles at 0–30 degrees) and −*δσ_av-down_* (*δσ_av_* for angles at 30–60 degrees). The non-uniform received incident wave amount is depicted by the effective incident factor *ω_eff_*, which is a normalized weight function (yellow curves in Figure 9). For instance, in the planar case, all the amount of incident wave can be received when *θ* ranges from −30–30 degrees, then the weight is set as 1. On the contrary, while the fibers are shaded in the yarn, namely, the incident angle *θ* is ± 90 degrees, no incident wave can reach these parts, and the corresponding weight is 0. In addition, the weights between these two limits are assumed to vary linearly.

The relative deviation of the effective absorption cross-section *δσ_eff_* is then the first-order moment of *δσ* weighted by *ω_eff_*, within the angle ranging between −90–90 degrees. Accordingly, the relation is formulated in Equation (1) as follows:

(1) $$\delta \sigma_{eff}=\int_{-90}^{90}\omega_{eff}\delta \sigma d\theta /\int_{-90}^{90}\omega_{eff}d\theta ,$$

to be more specific, it can be seen that the planar configuration presents *δσ_eff_* of 0%, the tip configuration exhibits *δσ_eff_* of 1/3 × *δσ_av-amp_*, and for the half-tip one, *δσ_eff_* is about 1/15 × *δσ_av-amp_*. When the probabilities for all the possible configurations are uniform, the average absorption cross-sections are approximately 2/15 × *δσ_av-amp_*. In such a case, the effective absorption cross-section for the triangular shape is about 1.9% larger than the circular shape. Simultaneously, considering the extracted mean value *δσ_av-full_* of 2.5% (as seen in Figure 8), the overall enhancement on *δσ_eff_* is thus approximately 4.4%.

From the above simulations and analyses, it was further verified that the triangular fiber can improve the overall FIR performance. The underlying mechanism for enhancement was also revealed. In such a physical process, the anisotropic geometry in a triangular fiber induces absorption fluctuation at different incident angles, though not always outperforming the circular fiber. The arrangement of tightly-aligned fibers further puts leverage on such a fluctuation, and contributes to the larger effective absorption of the yarn, thus, to ultimately enhancing the FIR performance in the triangular fibers.

## 4. Conclusions

In summary, this work shows a novel triangular-based PA fiber with a remarkable far-infrared performance in knitted full-needle fabrics. The improvement in the fabric far-infrared absorption property can be attributed to the triangular PA fibers, which by means of the shape-editing-emissivity method adjust the absorption cross-section, thereby impacting its emission property as well as the far-infrared performance. Meanwhile, the systemic simulation on both a single fiber and the aggregate of fibers in a yarn further confirmed that the triangular fiber is helpful in improving far-infrared performance without any functional additives. The possible enhancement mechanism of triangular fibers optimizing the far-infrared performance of non-additive fabrics is supposed to be due to the different rotation angle of the triangular fibers inside a single yarn, which can be adapted to receive more far-infrared incident waves from different directions at a relative higher efficiency. On the other hand, the received far-infrared radiation will be held inside the fiber longer to develop a larger optical path difference, hence, the triangular fiber possesses a higher emission ability of far-infrared radiation accordingly. Moreover, though the underlying mechanism still requires further understanding, this work presents great potential for high-performance and non-polluting far-infrared textiles, thus providing a new universal approach for improving non-additive FIR textiles.

## Figures and Tables

**Table 1:** The detail specifications of PA fibers and elastic yarns.

Type of Yarns	Detail Parameters			
PA fibers	Cross-section	Glossiness	Draft degree	Fineness
	Circular and triangular	Bright	FDY	44/34
Elastic yarns ^1^	Circular, full-dull and DTY yarns with 10 filaments			

^1^ Note that the elastic yarns are auxiliary yarns without far-infrared function. FDY = Fully drawn yarn, DTY = Draw textured yarn, 44/34 = 44dtex/34filaments.
